# ﻿*Lespedezajianghuensis* (Fabaceae), a new species from riparian meadows of Yangtze River basin, China

**DOI:** 10.3897/phytokeys.252.144564

**Published:** 2025-02-10

**Authors:** Song Huang, Mei-Qian Chen, Feng Song, Jia-Xiang Li, Bo Pan, Hui-Yi Zhong, Li-Qun Zhou, Ang Liu, Yu-Tao Zheng, Pan Zhao

**Affiliations:** 1 Central South University of Forestry and Technology, Changsha 410004, Hunan, China Central South University of Forestry and Technology Changsha China; 2 Xishuangbanna Tropical Botanical Garden, Chinese Academy of Sciences, Mengla 666303, Yunnan, China Chinese Academy of Sciences Mengla China; 3 Jiangxi Academy of Forestry Science, Nanchang 330032, Jiangxi, China Jiangxi Academy of Forestry Science Nanchang China

**Keywords:** Fabaceae, morphological traits, new species, taxonomy, wetland

## Abstract

*Lespedezajianghuensis* (Fabaceae), from Yangtze River basin, China, is described and illustrated as a new species. It is a dwarf shrublet occurring in the riparian meadows along the banks of rivers and lakes. It is morphologically similar to *L.cuneata*, *L.lichiyuniae*, and *L.caraganae*, but differs by its prostrate or decumbent habit, angulate but not furrowed stems, and small stipules (ca. 1 mm). Phylogenetic analyses based on combination of five chloroplasts fragments and ITS sequence confirmed that it belongs to Lespedezasect.Junceae. We also evaluate its conservation status as Least Concern (LC).

## ﻿Introduction

The genus *Lespedeza* Michx. (Fabaceae) is characterized by trifoliolate leaves, small pea-like flowers, and single-seeded pod (Huang et al. 2010). The genus was first described by Michaux (1803). It has a primarily East Asian distribution, with the majority of the ca. 40–50 recognized species found in China, Japan, Korea, and adjacent regions (Ohwi and Kitagawa 1983; Ohashi 2005; Ohashi et al. 2009a, 2009b). A few species occur in North America, where the genus likely originated and then dispersed to Asia in the past (Xu et al. 2012). Taxonomic delimitation within *Lespedeza* has been challenging, with ongoing debate about species boundaries and relationships (Ohashi 2005; Xu et al. 2011). Based on the results of distribution and molecular phylogenetic analyses, Ohashi and Nemoto (2014) re-circumscribed the subgenera, confining L.subg.Lespedeza to North America and L.subg.Macrolespedeza to Asia.

China is a major center of diversity for *Lespedeza*, harboring 27 species, including 14 endemic species (Huang et al. 2010). Recent field investigations and taxonomic studies have led to the discovery and description of new species from various regions of China, such as *L.jiangxiensis* Bo Xu bis, X. F. Gao & Li Bing Zhang from Jiangxi Province (Xu et al. 2013), *L.pseudomaximowiczii* D. P. Jin, Bo Xu bis & B. H. Choi from Anhui, Henan and Zhejiang Province (Jin et al. 2018) and *L.danxiaensis* Q. Fan, W.Y. Zhao & K.W. Jiang from Guangdong Province (Zhao et al. 2021). These findings highlight the need for continued exploration and taxonomic research on *Lespedeza* in China.

During a botanical survey in the middle and lower reaches of Yangtze River, from 2021 to 2023, we found an unknown *Lespedeza* plant in riparian meadows. It resembles *L.cuneata* in the white flowers, narrow leaflets with pinnate lateral veins, but differs by its procumbent habit and smaller stipules. After carefully examining specimens and literature, together with a molecular phylogenetic analysis based on combination of five plastid fragments and Internal Transcribed Spacers (ITS), we describe and illustrate it as a new species.

## ﻿Materials and methods

### ﻿Morphological study

The morphological characters were examined, based on the living plants and specimens deposited in the herbaria CSFI, HIB, HNNU, IBSC, JXCM, JXAU, NAS, and PE, herbarium acronyms as in Thiers (2021). Online databases such as CVH (https://www.cvh.ac.cn/), PPBC (http://ppbc.iplant.cn/) and JSTOR (https://www.jstor.org/) were also examined to document more records of the new species.

### ﻿Phylogenetic analyses

Two representative individuals from different populations were selected for molecular analyses, *Jia-Xiang Li et al. 2023101903* from Jiangxi Province, and *Song Huang et al. HD-25* from Hubei Province. A total of 29 accessions, representing 28 species of *Lespedeza*, two species of *Kummerowia* [*K.stipulacea* (Maxim.) Makino and *K.striata* (Thunb.) Schindl.] and three species of *Campylotropis* [*C.hirtella* (Franch.) Schindl., *C.macrocarpa* (Bunge) Rehder., and *C.polyantha* (Franch.) Schindl.], were sampled for outgroup comparison. Most sequences except the new species were downloaded from GenBank. Total genomic DNA was extracted from silica-gel-dried leaf material using the modified CTAB procedure (Doyle and Doyle 1987), with reference to the assembly of the original sequences by Xue et al. (2023). Phylogenetic reconstruction of the new species and related taxa was carried out using nuclear DNA internal transcribed spacer sequences (ITS) linked to five chloroplast loci (*rpl16*, *rpl32-trnL*, *rps16-trnQ*, *trnL-F*, *trnK/matK*), analyses method of combined chloroplast and ITS sequences data with reference to Xu et al. (2012). Taxa sampled and GenBank accession numbers for the six datasets are listed in Appendix 1. The phylogenetic relationships were estimated by generating a Maximum likelihood (ML) trees using RAxML-HPC2 (8.2.12) with the GTRGAMMA model, and the support for individual nodes in the phylogenetic tree was assessed with1,000 bootstrap replicates (Stamatakis 2014). The resulting tree was visualized in TreeGraph 2 (Stöver and Müller 2010).

## ﻿Results

### ﻿Molecular phylogenetics

**Figure 1.**
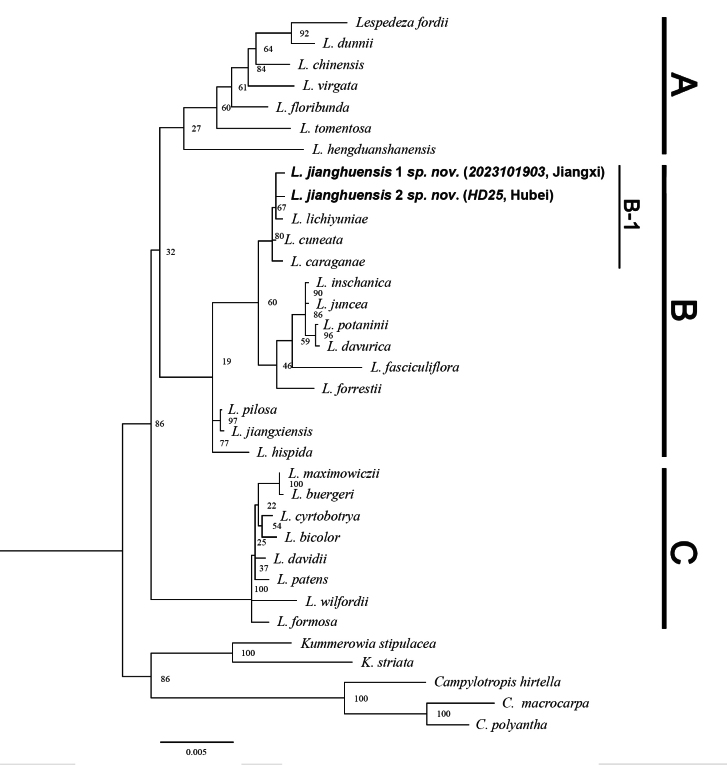
Phylogenetic relationships amongst 33 species of *Lespedeza*, *Kummerowia* and *Campylotropis* based on combined chloroplast and ITS sequences using Maximum Likelihood analysis. The new species in this study is indicated in boldface, with specimen collection numbers and provinces sampled indicated in parentheses. Numbers above branches indicate Maximum likelihood bootstrap values (before slash).

*Lespedeza* (BS = 86), *Kummerowia* (BS = 100), and *Campylotropis* (BS = 100) were all recovered as monophyletic in the phylogenetic tree of this study (Fig. 1). sect. Macrolespedeza taxa were clustered into a single clade (clade C) as sister to the sect. Junceae taxa, of which was divided into two clades (i.e., clade A and B) (BS = 32). The putative new species is deeply embedded in clade B and is considered to be a member of subclade B-1 consisting of *L.lichiyuniae* T. Nemoto, H. Ohashi & T. Itoh, *L.cuneata* (Dum. Cours.) G. Don, *L.caraganae* Bunge. (BS = 80, Fig. 1).

### ﻿Morphological comparison

Table 1 summarizes a detailed morphological comparison of the new species with three closely related species within subbranch B-1. These species have laterals veins to the margin of leaflets. Morphologically, the new species is most similar to *L.cuneata*, sharing features such as white flowers, and narrow leaflets. However, the new species differs from the latter by the procumbent habit, green or purplish black stems, sparse leaves along the branches, sparse hairs on the stems and lower side of leaflets, and short stipules ca. 1 mm (Table 1).

**Table 1.** Morphological comparison of *Lespedezajianghuensis* and its closely related species.

Character	*L.jianghuensis*	*L.cuneata*	*L.lichiyuniae*	*L.caraganae*
**Habit**	Prostrate or decumbent, up to 60 cm tall	Erect or ascending, up to 100 cm tall	Erect or ascending, up to 120 cm tall	Erect, up to 50 cm tall
**Stem**	Appressed- pubescent, subglabrous when old, green to purplish black, angular but not furrowed	Grayish green, densely ascending pubescent, multiple furrowed	Green to reddish brown, ascending- or appressed-pubescent, multiple furrowed	Grayish green, adpressed shortly hairy, multiple furrowed
**Leaflet**	Narrowly obovate, 5–16 × 2–4(–5) mm, glabrous above, adpressed hairy below	Cuneate or linear-cuneate, (7–)10–30 × 2–7 mm, subglabrous above, densely adpressed hairy below	Narrowly obovate, 4.6–28 × 1.6–7.7 mm, glabrous above, rather densely sericeous below	Oblong-linear, 20–40 × 2–4 mm, subglabrous above, adpressed hairy below
**Stipule**	Triangular, 0.7–1 mm	Lanceolate, 1–4 mm	Linearly triangular, 1–4 mm	Subulate, 2.5 mm
**Flower color**	White	White	Pale purple	White

### ﻿Taxonomic treatment

#### ﻿ Lespedeza jianghuensis

Taxon classificationPlantaeFabalesFabaceae

Song Huang, Jia X. Li & B. Pan bis sp. nov.

D7BA8BE6-ADF7-542E-A75E-02E712C5799E

urn:lsid:ipni.org:names:77356518-1

Figs 2–4

##### Type.

China • Jiangxi, Nanchang, Jinxian County, Qinglan Lake, 15–30 m a.s.l., 19 October 2023, *Jia-Xiang Li et al. 2023101903* (holotype: CSFI080349!, isotype HITBC!).

##### Diagnosis.

*L.jianghuensis* is morphologically most similar to *L.cuneata*, in having narrow leaflets and straight lateral veins to the margin, but differs from the latter by its prostrate or decumbent habit (vs. erect or ascending), stems strongly branched at the base (vs. stems simple), branches with appressed-pubescent (vs. densely ascending-pubescent), leaves sparse (vs. leaves crowded), leaflets narrowly obovate, terminal leaflets 5–16 mm long (vs. cuneate or linear-cuneate, terminal leaflets 7–30 mm long in *L.cuneata*), abaxial surfaces of leaves pubescent with obvious veins (vs. densely whitish pubescent, veins indistinct), stipules triangular, 0.7–1 mm long (vs. lanceolate, 1–4 mm long in *L.cuneata*) and calyx lobes narrowly triangular (vs. lanceolate) (Fig. 5).

**Figure 2.**
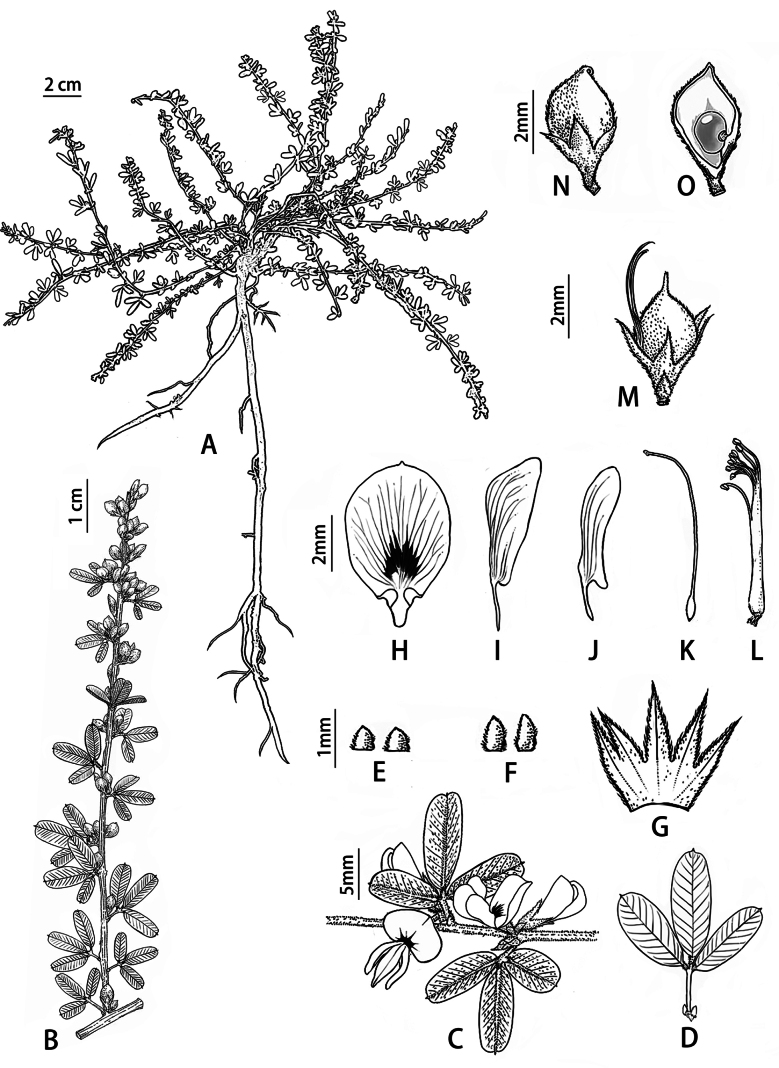
*Lespedezajianghuensis* Song Huang, Jia X. Li & B. Pan bis, sp. nov. **A** habit **B** fruiting branch **C** flowering branch **D** leaf **E** bracts **F** bracteoles **G** calyx **H** standard **I** keel-petal **J** wing **K** pistil **L** stamens **M** pod of chasmogamous flower **N** pod of cleistogamous flower **O** seed. Illustrated by Mei-Qian Chen.

##### Description.

**Figure 3.**
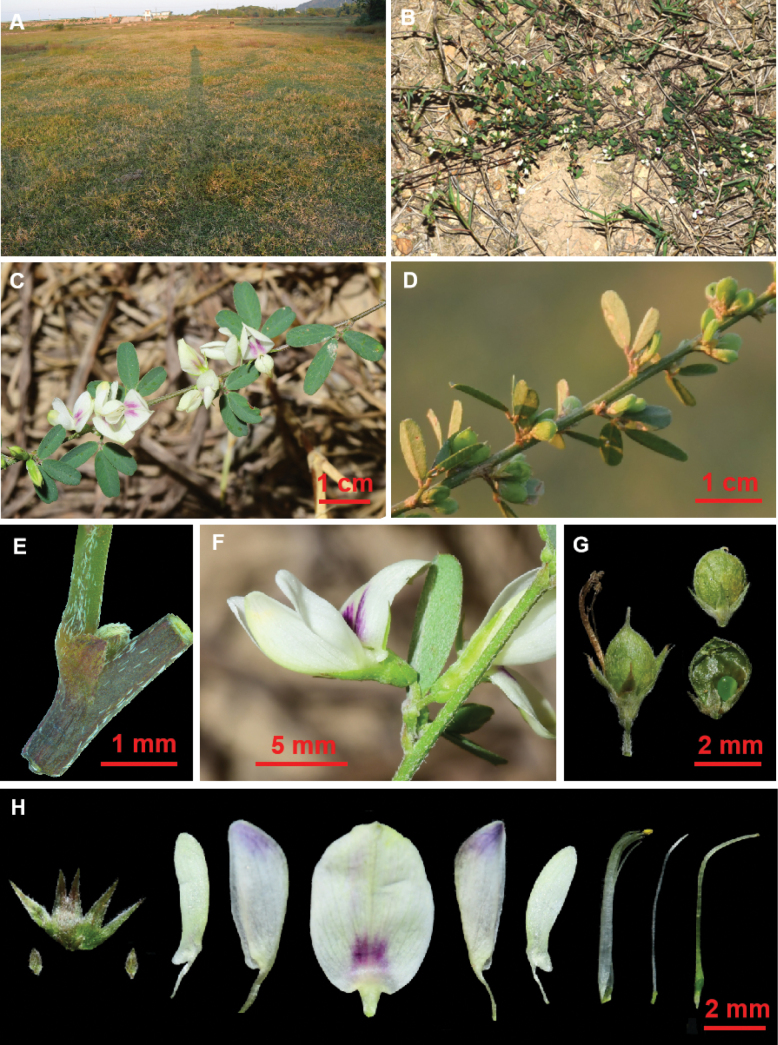
Morphology and habitat of *Lespedezajianghuensis* Song Huang, Jia X. Li & B. Pan bis, sp. nov. **A** habitat **B** habit **C** flowering branch **D** fruiting branch **E** stipule **F** lateral view of flower **G** fruit and seed, pod of chasmogamous flowers (left), pods of cleistogamous flowers (right) **H** flower dissections, from left to right (bracts and anatomical calyx; wing, keel-petal and standard; stamens and pistil). Photographed by Jia-Xiang Li and Song Huang.

Shrub, prostrate or procumbent, much branched at the base, up to 60 cm long (Fig. 3). Stem slightly angular, green when young, appressed white pubescent, purplish black when old, late glabrous. Trifoliolate; stipules persistent, triangular, 0.7–1 × 0.4–0.8 mm; petiole 1–6 mm long, appressed pubescent; pulvini 0.3–0.7 mm, appressed pubescent. Leaflets glabrous above, adpressed hairy beneath, late glabrous, pinnate veins clear on both side, (6–)8–14 pairs, reaching margin of leaflets, narrowly obovate, 5–16 × 2–4(–5) mm, apex obtuse or slightly emarginate, apiculate, terminal leaflets slightly larger than lateral ones; pulvinus 0.3–0.6 mm; rachis short, 0.5–1.0 mm. Racemes axillary, not exceeding the leaves, with 2–6 flowers, pedicels very short (shorter than 1 mm); bracts ovate-triangular, 0.3–0.6 mm; bracteoles 2 at base of calyx, ovate-triangular, 0.5–1 mm, equal to or shorter than calyx tube, appressed hairy. Flowers 5–9 mm; calyx campanulate, 2.5–4.5 mm, 5-lobed, lobes narrowly triangular, 1–2.8 mm, appressed hairy, margins ciliate, lateral and lowest calyx lobes lobed to below middle, upper lobes split about 1/3 of calyx; corolla white; standard oval, base with purple spot, reflexed at anthesis, 5.5–8 mm, with a claw and 2 auricles at base, lamina 5–7 × 4–4.5 mm, obtuse with a point at apex; wings narrowly elliptic, slightly shorter than the keel petals, 5–6.5 mm, lamina 4–6 × 1–2 mm, slightly auriculate at base, with a 1–1.5 mm claw; keel 5.5–8 mm, often tinged purple at apex, lamina 4–6.5 × 1–2.5 mm, narrowly obovate to obovate, obtuse at apex, abruptly narrowed to claw, claw ca. 1.5–2 mm.; diadelphous stamens (9+1), 6–7.5 mm; pistil, 6.5–8 mm, ovary obovate, glabrous; cleistogamous flowers clustered in the lower leaf axils of the stem, sessile or subsessile; Pods indehiscent, 1-seeded, densely adpressed hairy. Chasmogamous pods oval, 3–4.5 × 1.5–2.5 mm, with a straight beak at apex. Cleistogamous pods nearly round, 2.5–3.5 × 1.8–2.5 mm, 2–3 times longer than persistent calyx, with a curved beak at the apex, beak ca. 0.3 mm long,

**Figure 4.**
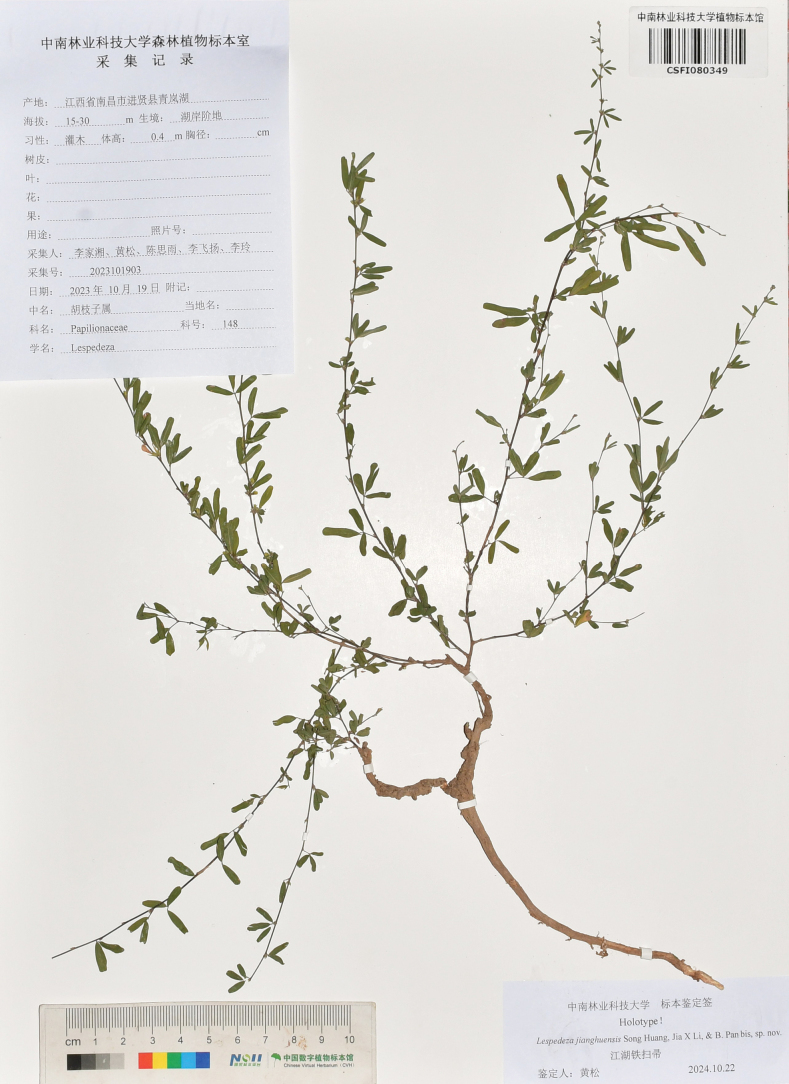
Holotype specimen of *Lespedezajianghuensis* Song Huang, Jia X. Li & B. Pan bis, sp. nov.

**Figure 5.**
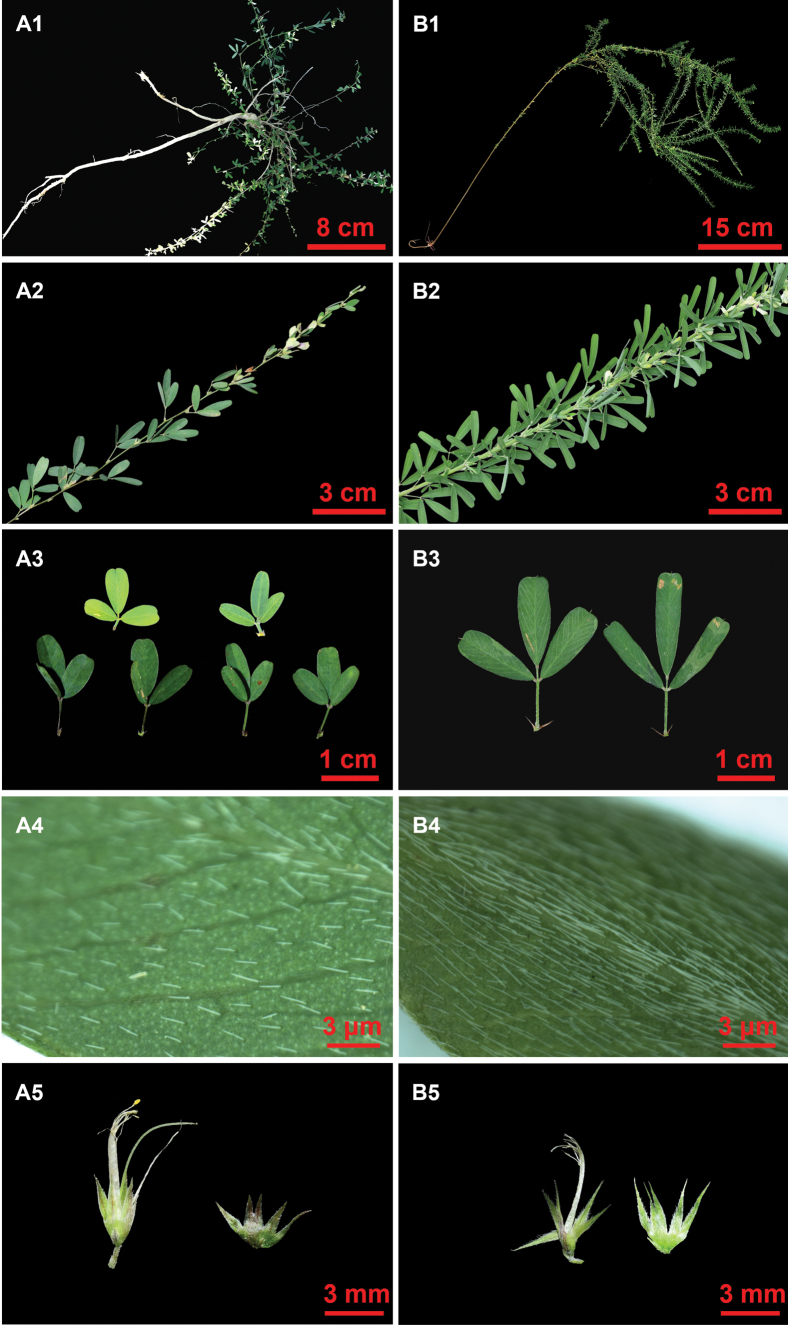
Morphological comparison between *Lespedezajianghuensis* Song Huang, Jia X. Li & B. Pan bis, sp. nov. (**A1–A5**) and *L.cuneata* (Dum. Cours.) G. Don (**B1–B5**) **A1, B1** plant habit **A2, B2** branches **A3, B3** adaxial view of leaf **A4, B4** abaxial surface of leaflet **A5, B5** calyx. Photographed by Song Huang.

##### Geographical distribution and habitat.

**Figure 6.**
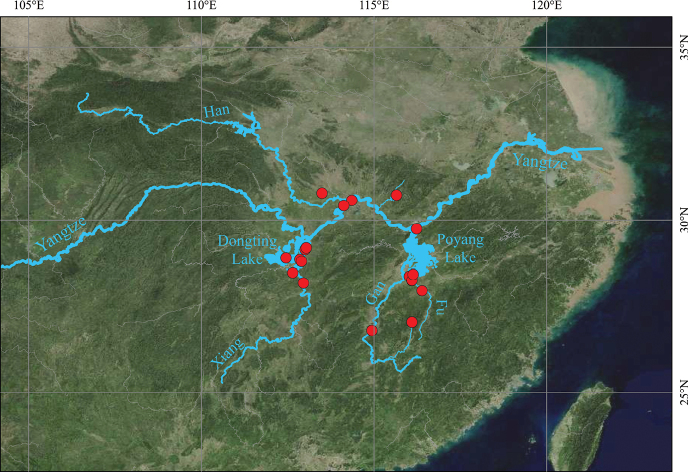
Geographic distribution (red dots) of *Lespedezajianghuensis* Song Huang, Jia X. Li, & B. Pan bis, sp. nov.

*Lespedezajianghuensis* is widely distributed in the middle reaches of Yangtze River basin in China (Fig. 6). such as Hunan, Jiangxi and Hubei Provinces. It grows on river floodplains, lakeshore terraces, mudflats, marshes, headlands, and other grasslands and wastelands close to rivers and lakes at elevation from 7 to 39 meters, and it is subject to seasonal flooding in summer.

##### Phenology.

Flowering September to October; fruiting October to November.

##### Etymology.

The specific epithet “*jianghuensis*” refers to the distribution area, with “jiang” standing for Yangtze River and its tributaries, and “hu” standing for the lakes in Yangtze River Basin.

##### Chinese name

**(assigned here).** jiāng hú tiě sào zhou (江湖铁扫帚).

##### Conservation status.

The new species is widely distributed along the Yangtze River, and it is likely to be more common and widespread than currently documented. It is therefore not considered to be in imminent conservation danger. The wild population of the new species is presumed to be around 50,000, and there is no known population size or population decline. It is evaluated as Least Concern (LC) according to IUCN Red List criteria (IUCN Standards and Petitions Committee 2022).

##### Additional specimens examined.

**China. Hubei**: Wuhan City, Wuchang District, Hesheng Bridge, 12 Jul. 1958, *Ying-Han Zhang 842* (HIB0041554). Yingcheng City, Yihe Township, Xier Village, 4 Oct. 2024, *Song Huang et al. HD-25* (HITBC); **Hunan**: Changsha city, Yuelu Mountain, along the Xiang River, 10 Aug. 1972, *Lin-Han Liu 8060* (HNNU00017822); Ningxiang City, Wangcheng District, Tuntou Lake, 5 Oct. 2024, *Song Huang et al. HD-26* (HITBC); Xiangyin County, Qingtan Township, 8 Aug. 2001, *Ke-Ming Liu & Guang-Wan Hu 24013* (HNNU00017820); Xiangyin County, Xianglu Mountain, 24 Oct. 2021, *Ang Liu & Guo-Hui Zhou DTH0298* (CSFI080351); Yueyang County, Lujiao Town, 26 Oct. 2021, *Ang Liu & Guo-Hui Zhou DTH0373* (CSFI080350); Yueyang County, Zhongzhou township, 21 Sep. 2024, *Song Huang et al. HD-05* (HITBC). **Jiangxi**: Fengcheng City, Dalan Town, Wangzhou Village, 1 Oct. 2024, *Song Huang et al. HD-20* (HITBC); Fengcheng City, Yuandu Town, Changyuan Village, 30 Sep. 2019, *Xiao-Lang Du 360981190930092LY* (JXCM0010153); Ganzhou City, Ningdu County, Xiaotian Township, 24 Oct. 1958, *Qi-Ming Hu 5622* (IBSC0180254); Ji’an City, Taihe County, by the Gan River, 15 Sep. 1980, *South Meadow group 297* (PE01791044); Jinxian County, Luoxi Township, Nanyang Village, 2 Oct. 2024, *Song Huang et al.* HD-21 (HITBC); Nanchang County, Wenshen Town, Cangtou Village, 2 Oct. 2024, *Song Huang et al. HD-24* (HITBC); Nanchang County, 28 Aug. 1940, *Migo H*. (NAS00387673, NAS00387675, NAS00387676); Yichun City, Tonggu County, Yingchao Village, 14 Oct. 1996, *Zheng-Ming Tao et al. 960460* (JXAU0007890).

## Supplementary Material

XML Treatment for Lespedeza jianghuensis

## ﻿Additional information

### Author ORCIDs

Song Huang https://orcid.org/0009-0009-1052-5840

Jia-Xiang Li https://orcid.org/0000-0003-0478-1409

Bo Pan https://orcid.org/0000-0002-0038-9664

Hui-Yi Zhong https://orcid.org/0009-0001-1003-1922

Li-Qun Zhou https://orcid.org/0009-0001-5425-9152

Ang Liu https://orcid.org/0000-0001-6281-7145

### Data availability

All of the data that support the findings of this study are available in the main text.

## ﻿Appendix 1

**Table A1.** GenBank accession numbers for the 28 species of *Lespedeza* and 5 outgroups.

Taxon	ITS	*rpl16*	*rpl32-trnL*	*rps16-trnQ*	*trnL-F*	*trnK/matK*
***Lespedeza* Michx.**						
*L.bicolor* Turcz.	JN402405	JN402497	JN402589	JN402683	JN402777	JN402872
*L.buergeri* Miq.	JN402406	JN402500	JN402590	JN402684	JN402778	JN402873
*L.caraganae* Bunge	JN402410	JN402502	JN402594	JN402688	JN402782	JN402877
*L.chinensis* G. Don	JN402416	JN402508	JN402600	JN402694	JN402788	JN402883
*L.cuneata* (Dum. Cours.) G. Don	JN402417	JN402509	JN402601	JN402695	JN402789	JN402884
*L.cyrtobotrya* Miq.	JN402422	JN402514	JN402606	JN402700	JN402794	JN402889
*L.davidii* Franch.	JN402428	JN402520	JN402612	JN402706	JN402800	JN402895
*L.davurica* (Laxm.) Schindl.	JN402425	JN402517	JN402609	JN402703	JN402797	JN402892
*L.dunnii* Schindl.	JN402431	JN402523	JN402615	JN402709	JN402803	JN402898
*L.fasciculiflora* Franch.	JN402432	JN402524	JN402616	JN402710	JN402804	JN402899
*L.floribunda* Bunge	JN402436	JN402528	JN402620	JN402714	JN402808	JN402903
*L.fordii* Schindl.	JN402440	JN402532	JN402624	JN402718	JN402812	JN402907
*L.formosa* (Vogel) Koehne	JN402443	JN402535	JN402627	JN402721	JN402815	JN402910
*L.forrestii* Schindl.	JN402448	JN402540	JN402632	JN402726	JN402820	JN402915
*L.hengduanshanensis* (C. J. Chen) Bo Xu bis, X. F. Gao & Li Bing Zhang	JN402434	JN402526	JN402618	JN402712	JN402806	JN402901
*L.inschanica* (Maxim.) Schindl.	JN402452	JN402544	JN402636	JN402730	JN402824	JN402919
*L.jianghuensis* Song Huang, Jia X. Li, & B. Pan bis (*2023101903*, Jiangxi)	PQ932036	PQ963378	PQ963378	PQ963378	PQ963378	PQ963378
*L.jianghuensis* Song Huang, Jia X. Li, & B. Pan bis (*HD25*, Hubei)	PQ932035	PQ963377	PQ963377	PQ963377	PQ963377	PQ963377
*L.jiangxiensis* Bo Xu bis, X. F. Gao & Li Bing Zhang	JN402455	JN402546	JN402639	JN402733	JN402827	JN402922
*L.juncea* (L. f.) Pers.	JN402457	JN402548	JN402641	JN402735	JN402829	JN402924
*L.lichiyuniae* T. Nemoto, H. Ohashi & T. Itoh	JN402461	JN402552	JN402645	JN402739	JN402833	JN402928
*L.maximowiczii* C. K. Schneid.	JN402464	JN402555	JN402648	JN402742	JN402836	JN402931
*L.patens* Nakai	JN402466	JN402557	JN402650	JN402744	JN402838	JN402933
*L.pilosa* (Thunb.) Siebold & Zucc.	JN402467	JN402558	JN402651	JN402745	JN402839	JN402934
*L.potaninii* Vassilcz.	JN402469	JN402560	JN402653	JN402747	JN402841	JN402936
*L.tomentosa* (Thunb.) Siebold ex Maxim.	JN402476	JN402565	JN402659	JN402753	JN402848	JN402943
*L.virgata* (Thunb.) DC.	JN402480	JN402569	JN402663	JN402757	JN402852	JN402947
*L.wilfordii* Ricker	JN402484	JN402573	JN402667	JN402761	JN402856	JN402951
*L.hispida* (Franch.) T. Nemoto & H. Ohashi	JN402450	JN402542	JN402634	JN402728	JN402822	JN402917
***Kummerowia* Schindl.**						
*K.striata* (Thunb.) Schindl.	MN192633	JN402583	JN402677	JN402771	JN402866	JN402961
*K.stipulacea* (Maxim.) Makino	JN402676	JN402582	JN402676	JN402770	JN402865	JN402960
***Campylotropis* Bunge**						
*C.macrocarpa* (Bunge) Rehder	MN721989	NC044100	NC044100	NC044100	NC044100	NC044100
*C.hirtella* (Franch.) Schindl.	JN402491	JN402580	JN402674	JN402768	JN402863	JN402958
*C.polyantha* (Franch.) Schindl.	LC740845	NC064519	NC064519	NC064519	NC064519	NC064519
